# (*E*)-4-Bromo-2-[(2,6-diisopropyl­phen­yl)imino­meth­yl]phenol

**DOI:** 10.1107/S1600536812021605

**Published:** 2012-05-19

**Authors:** P. Balamurugan, K. Kanmani Raja, S. Kutti Rani, G. Chakkaravarthi, G. Rajagopal

**Affiliations:** a Department of Chemistry, Government Arts College (Men), Nandanam, Chennai 600 035, India; b Department of Chemistry, Government Thirumagal Mills College, Gudiyattam 632 604, India; c Department of Chemistry, B.S. Abdur Rahman University, Vandalur, Chennai 600 049, India; d Department of Physics, CPCL Polytechnic College, Chennai 600 068, India; e Department of Chemistry, Government Arts College, Melur 625 106, India

## Abstract

In the title compound, C_19_H_22_BrNO, the dihedral angle between the benzene rings is 76.17 (14)° and an intra­molecular O—H⋯N hydrogen bond with an *S*(6) graph-set motif is present. One methyl group is disordered over two sets of sites with site occupancies of 0.66 (3) and 0.34 (3). A weak inter­molecular C—H⋯π inter­action is observed in the crystal structure.

## Related literature

For the biological activity of Schiff base ligands, see: Daier *et al.* (2004 ▶); Santos *et al.* (2001 ▶). For related structures, see: Raja *et al.* (2008 ▶); Lin *et al.* (2005 ▶).
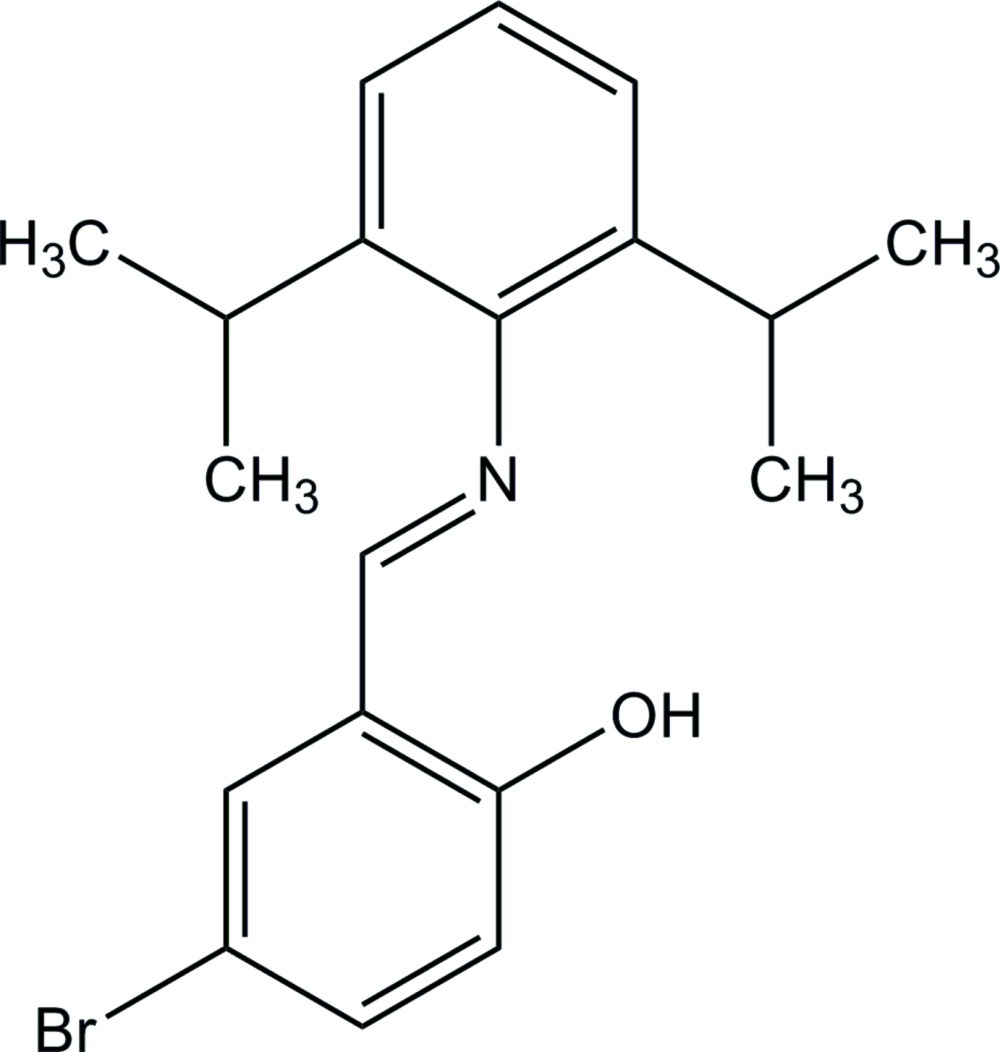


## Experimental

### 

#### Crystal data

C_19_H_22_BrNO
*M* *_r_* = 360.29
Orthorhombic, 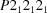

*a* = 6.1851 (12) Å
*b* = 12.759 (3) Å
*c* = 22.698 (5) Å
*V* = 1791.3 (6) Å^3^
*Z* = 4
Mo *K*α radiation
μ = 2.30 mm^−1^
*T* = 295 K
0.24 × 0.22 × 0.18 mm

#### Data collection

Bruker Kappa APEXII diffractometer
Absorption correction: multi-scan (*SADABS*; Sheldrick, 1996 ▶) *T*_min_ = 0.609, *T*_max_ = 0.683
23590 measured reflections
5107 independent reflections
3242 reflections with *I* > 2σ(*I*)
*R*_int_ = 0.039

#### Refinement

*R*[*F* ^2^ > 2σ(*F* ^2^)] = 0.040
*wR*(*F* ^2^) = 0.100
*S* = 1.01
5107 reflections
214 parameters
H-atom parameters constrained
Δρ_max_ = 0.38 e Å^−3^
Δρ_min_ = −0.22 e Å^−3^
Absolute structure: Flack (1983 ▶), 2179 Friedel pairs
Flack parameter: 0.011 (10)

### 

Data collection: *APEX2* (Bruker, 2004 ▶); cell refinement: *SAINT* (Bruker, 2004 ▶); data reduction: *SAINT*; program(s) used to solve structure: *SHELXS97* (Sheldrick, 2008 ▶); program(s) used to refine structure: *SHELXL97* (Sheldrick, 2008 ▶); molecular graphics: *PLATON* (Spek, 2009 ▶); software used to prepare material for publication: *SHELXL97*.

## Supplementary Material

Crystal structure: contains datablock(s) global, I. DOI: 10.1107/S1600536812021605/is5140sup1.cif

Structure factors: contains datablock(s) I. DOI: 10.1107/S1600536812021605/is5140Isup2.hkl

Supplementary material file. DOI: 10.1107/S1600536812021605/is5140Isup3.cml

Additional supplementary materials:  crystallographic information; 3D view; checkCIF report

## Figures and Tables

**Table 1:** Hydrogen-bond geometry (Å, °) *Cg*2 is the centroid of the C14–C19 ring.

*D*—H⋯*A*	*D*—H	H⋯*A*	*D*⋯*A*	*D*—H⋯*A*
O1—H1⋯N1	0.82	1.87	2.597 (3)	147
C16—H16⋯*Cg*2^i^	0.93	2.86	3.522 (3)	129

Symmetry code: (i) 
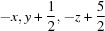
.

**Fig. 1.**
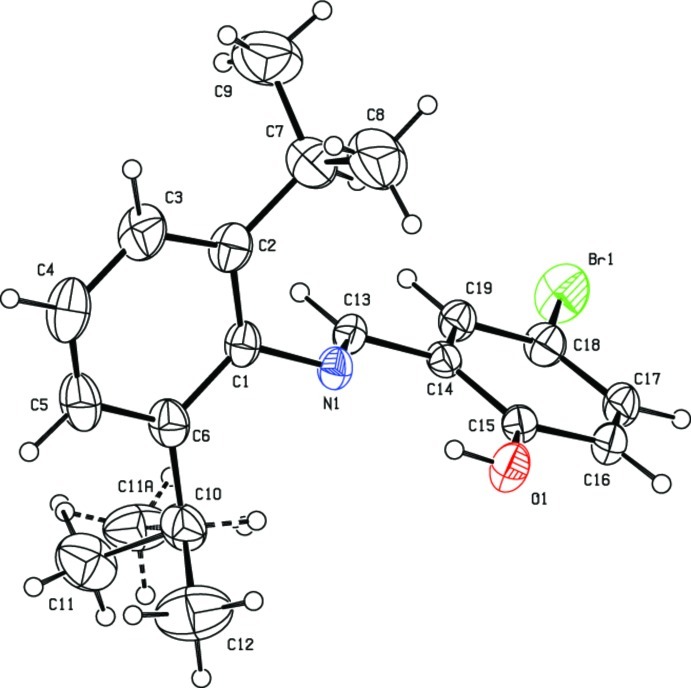
The molecular structure of the title compound, with atom labels and 30% probability displacement ellipsoids for non-H atoms.

## supplementary crystallographic information

### Comment

Schiff base derivatives are known to exhibit catalytic (Daier *et al.*, 2004), antibacterial, antitumor and antitoxic (Santos *et al.*, 2001) activities. The geometric parameters in the title compound are comparable with the similar reported structures (Raja *et al.*, 2008; Lin *et al.*, 2005). The dihedral angle between the aromatic rings (C1–C6) and (C14–C19) is 76.17 (14)°. One of the methyl groups in the molecule is disordered over two positions with site occupancies of 0.66 (3) and 0.34 (3). The molecule adopts an anti-periplanar [C1—N1—C13—C14 = -179.5 (2)°] conformation about the C=N double bond. The molecular structure is stabilized by an intramolecular O—H···N hydrogen bond and the crystal structure exhibit a weak intermolecular C—H···π (Table 1 ) interaction.

### Experimental

An ethanolic solution (10 ml) of 2,6-diisopropylaniline (2 mmol) was magnetically stirred in a round bottom flask followed by drop wise addition of ethanolic solution (10 ml) of 5-bromosalicylaldehyde (2 mmol). The reaction mixture was then refluxed for 3 h and upon cooling to 273 K, a yellow solid precipitated from the reaction mixture. The solid which separated out was filtered, washed with ice cold ethanol and dried over anhydrous CaCl_2_. Single crystal of good diffraction quality was obtained by the recrystallization of compound with the ethanol solution by slow evaporation method. Yield: 70%.

### Refinement

The site occupancies of disordered atoms were refined as C11/C11(A) = 0.66 (3)/0.34 (3). H atoms were positioned geometrically with C—H = 0.93–0.97 Å and O—H = 0.82 Å and allowed to ride on their parent atoms, with *U*_iso_(H) = 1.5*U*_eq_(O), 1.2*U*_eq_(C) or 1.5*U*_eq_(C_methyl_).

### Crystal data

**Table:**

C_19_H_22_BrNO	*F*(000) = 744
*M**_r_* = 360.29	*D*_x_ = 1.336 Mg m^−^^3^
Orthorhombic, *P*2_1_2_1_2_1_	Mo *K*α radiation, λ = 0.71073 Å
Hall symbol: P 2ac 2ab	Cell parameters from 2359 reflections
*a* = 6.1851 (12) Å	θ = 1.8–29.8°
*b* = 12.759 (3) Å	µ = 2.30 mm^−^^1^
*c* = 22.698 (5) Å	*T* = 295 K
*V* = 1791.3 (6) Å^3^	Prism, light yellow
*Z* = 4	0.24 × 0.22 × 0.18 mm

### Data collection

**Table:**

Bruker Kappa APEXII diffractometer	5107 independent reflections
Radiation source: fine-focus sealed tube	3242 reflections with *I* > 2σ(*I*)
Graphite monochromator	*R*_int_ = 0.039
ω and φ scans	θ_max_ = 29.8°, θ_min_ = 1.8°
Absorption correction: multi-scan (*SADABS*; Sheldrick, 1996)	*h* = −8→8
*T*_min_ = 0.609, *T*_max_ = 0.683	*k* = −17→11
23590 measured reflections	*l* = −30→31

### Refinement

**Table:**

Refinement on *F*^2^	Secondary atom site location: difference Fourier map
Least-squares matrix: full	Hydrogen site location: inferred from neighbouring sites
*R*[*F*^2^ > 2σ(*F*^2^)] = 0.040	H-atom parameters constrained
*wR*(*F*^2^) = 0.100	*w* = 1/[σ^2^(*F*_o_^2^) + (0.0449*P*)^2^ + 0.1641*P*] where *P* = (*F*_o_^2^ + 2*F*_c_^2^)/3
*S* = 1.01	(Δ/σ)_max_ < 0.001
5107 reflections	Δρ_max_ = 0.38 e Å^−^^3^
214 parameters	Δρ_min_ = −0.22 e Å^−^^3^
0 restraints	Absolute structure: Flack (1983), 2179 Friedel pairs
Primary atom site location: structure-invariant direct methods	Flack parameter: 0.011 (10)

### Fractional atomic coordinates and isotropic or equivalent isotropic displacement parameters (Å^2^)

**Table:**

	*x*	*y*	*z*	*U*_iso_*/*U*_eq_	Occ. (<1)
Br1	0.52689 (6)	0.49239 (2)	0.950698 (17)	0.07970 (14)	
O1	1.1558 (3)	0.14355 (16)	0.90753 (9)	0.0593 (5)	
H1	1.1027	0.0973	0.8870	0.089*	
N1	0.8534 (3)	0.04639 (16)	0.84992 (9)	0.0439 (5)	
C1	0.7847 (4)	−0.0403 (2)	0.81528 (12)	0.0454 (6)	
C2	0.6525 (5)	−0.1178 (2)	0.83940 (14)	0.0579 (7)	
C3	0.6048 (6)	−0.2032 (2)	0.80436 (16)	0.0747 (10)	
H3	0.5181	−0.2565	0.8192	0.090*	
C4	0.6846 (7)	−0.2105 (3)	0.74734 (17)	0.0814 (11)	
H4	0.6517	−0.2690	0.7246	0.098*	
C5	0.8084 (6)	−0.1344 (3)	0.72451 (16)	0.0736 (10)	
H5	0.8562	−0.1399	0.6858	0.088*	
C6	0.8666 (5)	−0.0472 (2)	0.75789 (13)	0.0542 (7)	
C7	0.5681 (6)	−0.1119 (2)	0.90207 (15)	0.0744 (10)	
H7	0.5856	−0.0393	0.9154	0.089*	
C8	0.7009 (9)	−0.1799 (5)	0.94217 (19)	0.1273 (19)	
H8A	0.6944	−0.2512	0.9288	0.191*	
H8B	0.6448	−0.1756	0.9815	0.191*	
H8C	0.8483	−0.1563	0.9418	0.191*	
C9	0.3341 (7)	−0.1387 (5)	0.9075 (2)	0.1205 (18)	
H9A	0.2865	−0.1250	0.9470	0.181*	
H9B	0.3135	−0.2115	0.8986	0.181*	
H9C	0.2519	−0.0968	0.8805	0.181*	
C10	1.0109 (5)	0.0367 (2)	0.73253 (13)	0.0632 (7)	
H10A	1.0195	0.0961	0.7598	0.076*	0.66 (3)
H10B	1.0424	0.0756	0.7688	0.076*	0.34 (3)
C11	0.933 (2)	0.0755 (10)	0.6705 (6)	0.097 (3)	0.66 (3)
H11A	0.7786	0.0838	0.6708	0.145*	0.66 (3)
H11B	0.9724	0.0249	0.6411	0.145*	0.66 (3)
H11C	0.9998	0.1415	0.6616	0.145*	0.66 (3)
C11A	0.884 (2)	0.114 (2)	0.7024 (16)	0.090 (8)	0.34 (3)
H11D	0.9780	0.1663	0.6856	0.135*	0.34 (3)
H11E	0.7874	0.1474	0.7299	0.135*	0.34 (3)
H11F	0.8016	0.0816	0.6717	0.135*	0.34 (3)
C12	1.2339 (6)	−0.0035 (5)	0.7194 (3)	0.1209 (16)	
H12A	1.2247	−0.0607	0.6920	0.181*	
H12B	1.3004	−0.0274	0.7552	0.181*	
H12C	1.3193	0.0518	0.7026	0.181*	
C13	0.7284 (4)	0.12241 (19)	0.86014 (11)	0.0422 (6)	
H13	0.5887	0.1212	0.8450	0.051*	
C14	0.7982 (4)	0.21236 (17)	0.89527 (10)	0.0384 (5)	
C15	1.0081 (4)	0.21757 (17)	0.91771 (10)	0.0422 (5)	
C16	1.0655 (4)	0.3034 (2)	0.95214 (12)	0.0512 (6)	
H16	1.2027	0.3063	0.9688	0.061*	
C17	0.9235 (4)	0.38342 (19)	0.96183 (12)	0.0524 (7)	
H17	0.9646	0.4406	0.9846	0.063*	
C18	0.7203 (5)	0.37927 (19)	0.93793 (12)	0.0503 (6)	
C19	0.6555 (4)	0.29447 (18)	0.90542 (11)	0.0451 (6)	
H19	0.5161	0.2919	0.8902	0.054*	

### Atomic displacement parameters (Å^2^)

**Table:**

	*U*^11^	*U*^22^	*U*^33^	*U*^12^	*U*^13^	*U*^23^
Br1	0.0866 (2)	0.04417 (15)	0.1083 (3)	0.00809 (16)	−0.00202 (19)	−0.02501 (15)
O1	0.0492 (10)	0.0627 (12)	0.0659 (13)	0.0044 (9)	−0.0119 (9)	−0.0171 (10)
N1	0.0484 (12)	0.0408 (10)	0.0425 (12)	0.0010 (9)	−0.0039 (10)	−0.0101 (9)
C1	0.0501 (14)	0.0390 (12)	0.0471 (15)	0.0059 (10)	−0.0079 (12)	−0.0103 (11)
C2	0.0687 (19)	0.0389 (13)	0.0662 (19)	−0.0029 (13)	−0.0054 (16)	−0.0082 (13)
C3	0.098 (3)	0.0426 (15)	0.083 (2)	−0.0111 (16)	−0.005 (2)	−0.0158 (16)
C4	0.107 (3)	0.0502 (17)	0.087 (3)	−0.0003 (18)	−0.020 (2)	−0.0313 (17)
C5	0.094 (3)	0.069 (2)	0.058 (2)	0.0120 (19)	−0.0027 (19)	−0.0291 (17)
C6	0.0587 (16)	0.0519 (14)	0.0520 (17)	0.0123 (13)	−0.0015 (14)	−0.0127 (13)
C7	0.108 (3)	0.0476 (15)	0.067 (2)	−0.0152 (18)	0.014 (2)	−0.0056 (15)
C8	0.107 (3)	0.197 (6)	0.078 (3)	−0.001 (4)	−0.007 (3)	0.025 (3)
C9	0.091 (3)	0.148 (5)	0.123 (4)	0.008 (3)	0.032 (3)	0.000 (3)
C10	0.0646 (18)	0.0675 (16)	0.0575 (16)	0.0092 (15)	0.0088 (15)	−0.0125 (13)
C11	0.099 (6)	0.106 (6)	0.085 (6)	0.019 (5)	0.003 (5)	0.024 (5)
C11A	0.070 (8)	0.087 (11)	0.114 (18)	0.003 (7)	0.011 (8)	0.043 (12)
C12	0.084 (2)	0.109 (3)	0.170 (5)	0.012 (3)	0.032 (3)	0.001 (4)
C13	0.0442 (14)	0.0415 (12)	0.0408 (15)	−0.0048 (11)	−0.0065 (11)	−0.0051 (11)
C14	0.0465 (13)	0.0362 (11)	0.0326 (12)	−0.0048 (10)	0.0028 (10)	−0.0024 (10)
C15	0.0466 (14)	0.0435 (11)	0.0364 (12)	−0.0054 (12)	−0.0005 (12)	−0.0017 (9)
C16	0.0529 (14)	0.0573 (14)	0.0435 (14)	−0.0130 (11)	−0.0068 (13)	−0.0046 (12)
C17	0.0686 (18)	0.0429 (12)	0.0458 (15)	−0.0169 (12)	−0.0014 (13)	−0.0108 (11)
C18	0.0628 (16)	0.0340 (11)	0.0541 (17)	−0.0041 (11)	0.0033 (13)	−0.0071 (11)
C19	0.0499 (14)	0.0396 (12)	0.0457 (14)	−0.0035 (11)	−0.0014 (12)	−0.0061 (11)

### Geometric parameters (Å, º)

**Table:**

Br1—C18	1.897 (3)	C10—C11A	1.438 (13)
O1—C15	1.334 (3)	C10—C12	1.502 (5)
O1—H1	0.8200	C10—C11	1.569 (9)
N1—C13	1.262 (3)	C10—H10A	0.9800
N1—C1	1.422 (3)	C10—H10B	0.9800
C1—C2	1.394 (4)	C11—H11A	0.9600
C1—C6	1.401 (4)	C11—H11B	0.9600
C2—C3	1.381 (4)	C11—H11C	0.9600
C2—C7	1.517 (5)	C11A—H11D	0.9600
C3—C4	1.388 (5)	C11A—H11E	0.9600
C3—H3	0.9300	C11A—H11F	0.9600
C4—C5	1.340 (5)	C12—H12A	0.9600
C4—H4	0.9300	C12—H12B	0.9600
C5—C6	1.393 (4)	C12—H12C	0.9600
C5—H5	0.9300	C13—C14	1.462 (3)
C6—C10	1.508 (4)	C13—H13	0.9300
C7—C9	1.492 (6)	C14—C19	1.389 (3)
C7—C8	1.502 (6)	C14—C15	1.396 (4)
C7—H7	0.9800	C15—C16	1.392 (3)
C8—H8A	0.9600	C16—C17	1.364 (4)
C8—H8B	0.9600	C16—H16	0.9300
C8—H8C	0.9600	C17—C18	1.370 (4)
C9—H9A	0.9600	C17—H17	0.9300
C9—H9B	0.9600	C18—C19	1.370 (3)
C9—H9C	0.9600	C19—H19	0.9300
			
C15—O1—H1	109.5	C12—C10—H10A	109.9
C13—N1—C1	121.1 (2)	C6—C10—H10A	109.9
C2—C1—C6	122.2 (3)	C11—C10—H10A	109.9
C2—C1—N1	120.6 (2)	C11A—C10—H10B	99.1
C6—C1—N1	117.1 (2)	C12—C10—H10B	99.0
C3—C2—C1	117.3 (3)	C6—C10—H10B	99.0
C3—C2—C7	120.4 (3)	C10—C11—H11A	109.5
C1—C2—C7	122.3 (2)	C10—C11—H11B	109.5
C2—C3—C4	120.9 (3)	C10—C11—H11C	109.5
C2—C3—H3	119.5	C10—C11A—H11D	109.5
C4—C3—H3	119.5	C10—C11A—H11E	109.5
C5—C4—C3	121.0 (3)	H11D—C11A—H11E	109.5
C5—C4—H4	119.5	C10—C11A—H11F	109.5
C3—C4—H4	119.5	H11D—C11A—H11F	109.5
C4—C5—C6	121.0 (3)	H11E—C11A—H11F	109.5
C4—C5—H5	119.5	C10—C12—H12A	109.5
C6—C5—H5	119.5	C10—C12—H12B	109.5
C5—C6—C1	117.6 (3)	H12A—C12—H12B	109.5
C5—C6—C10	120.8 (3)	C10—C12—H12C	109.5
C1—C6—C10	121.6 (2)	H12A—C12—H12C	109.5
C9—C7—C8	110.3 (4)	H12B—C12—H12C	109.5
C9—C7—C2	113.6 (3)	N1—C13—C14	121.5 (2)
C8—C7—C2	110.6 (3)	N1—C13—H13	119.2
C9—C7—H7	107.3	C14—C13—H13	119.2
C8—C7—H7	107.3	C19—C14—C15	119.6 (2)
C2—C7—H7	107.3	C19—C14—C13	119.7 (2)
C7—C8—H8A	109.5	C15—C14—C13	120.7 (2)
C7—C8—H8B	109.5	O1—C15—C16	118.7 (2)
H8A—C8—H8B	109.5	O1—C15—C14	122.7 (2)
C7—C8—H8C	109.5	C16—C15—C14	118.6 (2)
H8A—C8—H8C	109.5	C17—C16—C15	121.0 (2)
H8B—C8—H8C	109.5	C17—C16—H16	119.5
C7—C9—H9A	109.5	C15—C16—H16	119.5
C7—C9—H9B	109.5	C16—C17—C18	119.8 (2)
H9A—C9—H9B	109.5	C16—C17—H17	120.1
C7—C9—H9C	109.5	C18—C17—H17	120.1
H9A—C9—H9C	109.5	C19—C18—C17	120.8 (2)
H9B—C9—H9C	109.5	C19—C18—Br1	119.9 (2)
C11A—C10—C12	130.0 (11)	C17—C18—Br1	119.25 (19)
C11A—C10—C6	110.4 (6)	C18—C19—C14	120.0 (3)
C12—C10—C6	112.1 (3)	C18—C19—H19	120.0
C12—C10—C11	102.2 (7)	C14—C19—H19	120.0
C6—C10—C11	112.6 (4)		
			
C13—N1—C1—C2	−78.3 (3)	C1—C6—C10—C11A	−91.3 (18)
C13—N1—C1—C6	105.7 (3)	C5—C6—C10—C12	−65.0 (4)
C6—C1—C2—C3	0.3 (4)	C1—C6—C10—C12	115.6 (4)
N1—C1—C2—C3	−175.6 (3)	C5—C6—C10—C11	49.7 (8)
C6—C1—C2—C7	178.4 (3)	C1—C6—C10—C11	−129.7 (8)
N1—C1—C2—C7	2.6 (4)	C1—N1—C13—C14	−179.5 (2)
C1—C2—C3—C4	−0.5 (5)	N1—C13—C14—C19	179.9 (2)
C7—C2—C3—C4	−178.7 (3)	N1—C13—C14—C15	1.2 (4)
C2—C3—C4—C5	−0.6 (6)	C19—C14—C15—O1	−177.2 (2)
C3—C4—C5—C6	2.1 (6)	C13—C14—C15—O1	1.5 (4)
C4—C5—C6—C1	−2.2 (5)	C19—C14—C15—C16	2.9 (3)
C4—C5—C6—C10	178.3 (3)	C13—C14—C15—C16	−178.4 (2)
C2—C1—C6—C5	1.1 (4)	O1—C15—C16—C17	177.2 (2)
N1—C1—C6—C5	177.1 (3)	C14—C15—C16—C17	−2.8 (4)
C2—C1—C6—C10	−179.5 (3)	C15—C16—C17—C18	0.6 (4)
N1—C1—C6—C10	−3.5 (4)	C16—C17—C18—C19	1.5 (4)
C3—C2—C7—C9	−46.7 (5)	C16—C17—C18—Br1	−178.9 (2)
C1—C2—C7—C9	135.2 (4)	C17—C18—C19—C14	−1.4 (4)
C3—C2—C7—C8	78.0 (4)	Br1—C18—C19—C14	178.98 (19)
C1—C2—C7—C8	−100.1 (4)	C15—C14—C19—C18	−0.8 (4)
C5—C6—C10—C11A	88.1 (18)	C13—C14—C19—C18	−179.5 (2)

### Hydrogen-bond geometry (Å, º)

*Cg*2 is the centroid of the C14–C19 ring.

**Table:**

*D*—H···*A*	*D*—H	H···*A*	*D*···*A*	*D*—H···*A*
O1—H1···N1	0.82	1.87	2.597 (3)	147
C16—H16···*Cg*2^i^	0.93	2.86	3.522 (3)	129

Symmetry code: (i) −*x*, *y*+1/2, −*z*+5/2.
